# Comparison and correlations between findings of hysteroscopy and vaginal color Doppler ultrasonography for detection of uterine abnormalities in patients with recurrent implantation failure

**DOI:** 10.1515/med-2025-1226

**Published:** 2025-06-13

**Authors:** Nazli Navali, Esmat Sadat Kazemi, Hosein Azizi, Parvin Hakimi, Elham Eghbali

**Affiliations:** Women’s Reproductive Health Research Center, Tabriz University of Medical Sciences, Tabriz, Iran; Department of Obstetrics and Gynecology, Alzahra Hospital, Women s Reproductive Health Research Center, Tabriz University of Medical Sciences, Tabriz, Iran; Research Center of Psychiatry and Behavioral Sciences, Tabriz University of Medical Sciences, Tabriz, Iran; Medical Radiation Sciences Research Center, Tabriz University of Medical Sciences, Tabriz, Iran; Sarab Faculty of Medical Sciences, Sarab, Iran

**Keywords:** hysteroscopy, vaginal color Doppler ultrasonography, *in vitro* fertilization, recurrent implantation failure, IVF

## Abstract

**Background and aim:**

Hysteroscopy is the gold standard for diagnosing endometrial and cervical canal pathology and can be used alone or in combination with other methods. The study aimed to compare abnormal findings of vaginal color Doppler ultrasonography (transvaginal sonography [TVS]) and hysteroscopy.

**Methods:**

This study involved 100 infertile women with a history of two or more failed implantations of *in vitro* fertilization (IVF) from January 2020 to January 2021 in the infertility ward of Al-Zahra Hospital in Tabriz, Iran. All patients underwent hysteroscopy and TVS in the follicular phase to examine the endometrial cavity. We calculated the sensitivity and agreement of TVS color Doppler compared to hysteroscopy and using appropriate statistical tests.

**Results:**

The overall proportion of abnormal findings was 69 and 66% through hysteroscopy and TVS. The sensitivity and kappa statistics of TVS compared to hysteroscopy were 95.6 and 93.2%, respectively. Endometrial polyps were the most common abnormalities in hysteroscopy (31%) and TVS (25%). In examining the relationship between hysteroscopy findings and vaginal color Doppler ultrasonography findings, a significant association was found between submucosal myoma and non-homogeneous myometrium (OR = 1.8 (1.02–5.3); *P* = 0.027), endometrial polyps and non-homogeneous myometrium (OR = 2.7 (1.04–7.4); *P* = 0.025), intrauterine adhesion and uterine artery PI (OR = 1.9 (1.3–8.2); *P* = 0.001), endometrial atrophy and endometrial thickness (OR = 2.4 (1.01–4.5); *P* = 0.034), and thick/irregular endometrium and adenomyosis (OR = 2.5 (1.4–15.9); *P* = 0.001).

**Conclusion:**

Abnormal findings of hysteroscopy and TVS were observed more in patients who have a history of two or more unsuccessful IVFs. Comparing and evaluating the relationship between them can be considered a positive prognostic factor and a better diagnosis for achieving pregnancy in the IVF procedure in women with a history of recurrent implantation failure.

## 1 Introduction

Recurrent implantation failure (RIF), defined as two or more unsuccessful cycles of *in vitro* fertilization (IVF) embryo transfer, is challenging for clinicians and distressing for patients [1,2]. Despite technological advances in medicine, only about a quarter of IVF cycles lead to live births, and most patients remain infertile even after multiple IVF attempts. It has been reported that the infertility rate in different countries is between 5 and 30% [3]. A study conducted by Abangah et al. showed that the overall infertility rate in Iran is 10.9% [4]. Infertility can be attributed to fetal factors or uterine abnormalities. Several interventions have been suggested to improve the outcome of IVF after several failed attempts, only a few of which are evidence-based [1,5]. Therefore, first of all, these failure factors in IVF and fertility should be recognized.

Uterine abnormalities can be diagnosed by vaginal sonography, hysteroscopy, sonohysterography, and hysterosalpingography [6,7]. Hysteroscopy is mainly performed in cases of recurrent IVF failure and suspected intrauterine pathology because abnormalities of the uterine cavity can be easily evaluated with hysteroscopy. Therefore, it is considered the gold standard and definitive test in the evaluation of the uterine cavity [8]. In combination with hysteroscopy, vaginal color Doppler ultrasound is a non-invasive and easy imaging method with excellent clinical utility in the diagnosis of endometrial receptivity, which has recently attracted more attention [9,10]. This provides a unique tool for evaluating endometrial receptivity by virtue of its ability to monitor endometrial thickness, endometrial peristalsis wave, endometrial typing, and sub-endometrial blood flow parameters [11,12,13]. Considering that in patients with RIF with a long history of infertility, the comparison and relationship of hysteroscopy findings with vaginal color Doppler ultrasonography are poorly understood. Therefore, the present study aimed to compare correlations between the abnormal findings of hysteroscopy and vaginal color Doppler ultrasonography (TVS). It may be useful in predicting and improving the diagnostic power of abnormal findings in these patients.

## 2 Materials and methods

### 2.1 Study design and setting

The present study retrospectively was performed on 100 infertile women (between 20 and 41 years old) with a history of RIF from the beginning of January 2020 to the end of January 2021 in the infertility ward of Al-Zahra Educational and Treatment Hospital in Tabriz. They were infertile women due to various factors and underwent hysteroscopy and TVS color Doppler to assess the endometrial cavity in the follicular cycle by the same operator in our unit. The inclusion criteria for the study were as follows: Women aged 42 years and younger, not taking any specific medication for the past month, suffering from primary or secondary infertility, having a history of at least two unsuccessful transfers despite transferring at least one fresh or frozen embryo of good quality. Exclusion criteria included: (1) Smoking and alcohol consumption. (2) Having various diseases such as diabetes, cardiovascular, cancer, liver, thyroid, digestive, renal, hypertension, and rheumatoid arthritis. Based on the study type, we examined 100 patients who completed the steps. The sample size was set at 100 subjects based on the agreement percentage of hysteroscopy and TVS (0.77) based on a previous study [14] type I error (*α* = 0.05), Power = 0.8, and 10% compensation for poor information and accurate estimation. A total of 146 infertile women were referred to the infertility unit of Al-Zahra Hospital during the study. Of these, 100 subjects were included based on the study’s eligibility criteria. All patients signed an informed consent form before the study and treatment. The study has been approved by the Ethics Committee of Tabriz University of Medical Sciences (No: IR.TBZMED.REC.1400.954).

### 2.2 Technique of diagnostic hysteroscopy

Before the procedure, all patients underwent TVS color Doppler, and all abnormal findings were recorded. Then, hysteroscopy was performed under general anesthesia using a surgical hysteroscope (Carl Storz, Tatlinger, Germany). The uterine distention was performed by the glycine and using an electronic pump (Hysteromat; Karl Storz). Surgically assisted video hysteroscopy was performed using a monopolar electric resectoscope with an outer diameter of 9 mm. In patients with uterine pathology, appropriate treatment was performed simultaneously. In case of adhesion, polyp, or submucosal myoma, immediate adhesion lysis or hysteroscopic removal was performed. The septa were removed with a resectoscope or scissors. Cervical dilation was performed in patients with polyps larger than 2 cm or submucous myoma. If necessary, endometrial tissue samples were taken after examination of the cervical canal and uterine cavity, and then all samples were fixed in formaldehyde and sent for pathology evaluation.

### 2.3 Vaginal color Doppler ultrasonography

The same ultrasound device (Fukuda Denshi Co., Ltd., Tokyo, Japan) was used for this study, and the same physician performed all procedures. The vaginal ultrasound probe (8 MHz) was used for all subjects. TVS color Doppler was performed vaginally, and sonographic indexes of uterine vessels were reported as pulsatility index (PI), endometrial thickness, end-diastolic blood flow, and endometrial blood supply by mentioning Zone I, Zone II, Zone III, and sub-endometrium.

### 2.4 Statistical analysis

This study was analyzed using descriptive statistical methods and SPSS software (version 21.0, Chicago, IL, USA). Measurement data were expressed as mean ± standard deviation (mean ± SD) or number and percentage. The McNemar test was used to compare the abnormal findings of hysteroscopy and ultrasonography, and the agreement of the two methods to detect abnormal uterine findings was calculated using the kappa statistics. The relationship between abnormal findings of hysteroscopy and vaginal color Doppler ultrasonography was done using the Chi-square or Fisher exact test when the expected frequency of 2 by 2 table was less than 5, at least in a cell. The sensitivity of TVS was calculated compared to hysteroscopy (as a gold standard). Sensitivity is the ability of TVS to detect abnormalities in comparison with hysteroscopy. A *P*-value less than 0.05 was considered statistically significant.

## 3 Results

In this study, 100 infertile women who met our inclusion criteria were examined to determine hysteroscopy and TVS findings and their relationship. Patients’ demographic characteristics, including mean age (years), mean duration of infertility (years), mean number of failed IVF cycles, and cause of infertility, are presented in Table 1. Most patients showed some degree of abnormal uterine findings on hysteroscopy (69%) and ultrasonography (66%), while about one-third of the population had no obvious abnormal findings. Some abnormal findings of the uterus with hysteroscopy and TVS and their comparison are shown in Table 2. These findings include endometrial polyp, intrauterine adhesions, uterine septa, submucosal myoma, arcuate uterus, thick/irregular endometrium, and adenomyosis. The sensitivity and kappa statistics of TVS compared to hysteroscopy were 95.6 and 93.2%, respectively. The high agreement (100%) and sensitivity (100%) were estimated for submucosal myoma, while the minimum agreement and sensitivity were for uterine septa at 74.5 and 62.5%, respectively (Table 2).

**Table 1:** Characteristics of the participants

No. of women	100
Age (years)	34.32 ± 5.13
Duration of infertility (years)	9
Number of failed IVF cycles	2.56
Etiology of infertility	
Male factor	30 (0%)
Ovulatory disorder	21 (21%)
Tubal/peritoneal factor	9 (9%)
Endometriosis	13 (13%)
Combined factors	2 (2%)
Unexplained	25 (25%)

**Table 2:** Comparison of the findings of transvaginal sonography (TVS) and hysteroscopy

Findings	Hysteroscopy (*n* = 100)	TVS (*n* = 100)	kappa (%)	Sensitivity
Normal findings	31 (31)	34 (34)	—	—
Abnormal findings	69 (69)	66 (66)	93.2	95.65
Endometrial polyp	31 (31)	25 (25)	80.2	80.64
Intrauterine adhesions	10 (10)	0 (0)	0	90.3
Uterine septa	8 (8)	5 (5)	75.4	62.5
Submucosal myoma	6 (6)	6 (6)	100	100
Arcuate uterus	6 (6)	4 (4)	79	80.8
Thick/irregular endometrium	10 (10)	8 (8)	80.8	—
Adenomyosis	0 (0)	11 (11)	44.5	0
Total	71 (71%)	59 (59%)		

All patients were sampled after hysteroscopy to determine the pathological findings. 73% of the patients showed abnormal hysteroscopy pathological findings, and 27% had normal findings. Pathological findings, including endometrial polyp (17%), anovulatory endometrium (27%), disordered proliferative endometrium (29%), leiomyoma (8%), and endometrial atrophy (14%), were observed (Table 3).

**Table 3:** Distribution of hysteroscopy pathology findings

Pathology findings	No. of cases (%)
Normal pathology findings	27 (27)
Abnormal pathology detected	73 (73)
Endometrial polyp	17 (17)
Anovulatory endometrium	27 (27)
Disordered proliferative endometrium	29 (29)
Leiomyoma	8 (8)
Atrophic endometrium	14 (14)

Before performing hysteroscopy, a vaginal color Doppler ultrasonography was done for all patients to evaluate the relation with hysteroscopy findings. These parameters include (endometrial thickness, endometrial pattern, myometrial echogenicity, uterine artery PI, end-diastolic blood flow, and endometrial vascularity parameters in women), which are mentioned in Table 4.

**Table 4:** Findings of the uterine scoring system (vaginal color Doppler ultrasonography)

Parameter	No. of cases (%)
Endometrial thickness	
<7 mm	60 (60%)
>7 mm	40 (40%)
Endometrial pattern	
3 layers	46 (46%)
Others	54 (54%)
Myometrial echogenicity	
Homogeneous	49 (49%)
Non-homogeneous	51 (51%)
Uterine artery PI	
PI < 3	50 (50%)
PI > 3	50 (50%)
End diastolic blood flow	
Present	49 (49%)
Absent	51 (51%)
Endometrial vascularity	
Zone 3	16 (16%)
Zone 2	39 (39%)
Zone 1	38 (38%)
Subendometrium	7 (7%)

PI, pulsatility index.

In this study, there was a significant relation between the report of non-homogeneous myometrium in TVS color Doppler and the presence of submucosal myoma and uterine polyps in hysteroscopy, respectively (*P*-value = 0.027 and *P*-value = 0.025). In other words, all people (*n* = 6) with submucosal myoma and most (21 out of 31) with uterine polyps had non-homogeneous myometrium. In addition, a significant relation was observed between uterine artery PI (PI > 3) in TVS color Doppler and uterine adhesion in hysteroscopy (*P*-value = 0.001). All subjects with uterine adhesions (*n* = 10) had uterine artery PI > 3 on vaginal color Doppler ultrasonography. And, a significant relation was observed between uterine atrophy in hysteroscopy and endometrial thickness less than 7 mm in TVS color Doppler (*P*-value = 0.02). In other words, most people with uterine atrophy (12 out of 14 cases) had endometrial thickness less than 7 mm. Also, a significant relation was observed between thick/irregular endometrium in hysteroscopy and adenomyosis in TVS color Doppler (*P*-value = 0.001); most people with Thick/irregular endometrium had adenomyosis (Table 5). However, there was no relation between other hysteroscopic findings and TVS color Doppler findings (data not shown).

**Table 5:** Relation between the findings of vaginal color Doppler ultrasonography and the findings of hysteroscopy of the uterus

TVS findings	Hysteroscopy findings		*P*-value*
	**Submucosal myoma**		
Myometrial	Yes (*N* = 6)	No (*N* = 94)	1.8 (1.02–5.3)
Homogeneous (*N* = 49)	0 (0%)	49 (100%)	
Non-homogeneous (*N* = 51)	6 (11.8%)	45 (88.2%)	0.027
	**Endometrial polyp**		
Myometrial	Yes (*N* = 31)	No (*N* = 69)	2.7 (1.04–7.4)
Homogeneous (*N* = 49)	10 (20.4%)	39 (79.6%)	
Non-homogeneous (*N* = 51)	21 (41.2%)	30 (58.8%)	0.025
	**Intrauterine adhesions**		
Uterine artery PI	Yes (*N* = 10)	No (*N* = 90)	1.9 (1.3–8.2)
PI < 3 (*N* = 50)	0 (0%)	50 (100%)	
PI > 3 (*N* = 50)	10 (20%)	40 (80%)	0.001
	**Atrophic endometrium**		
Endometrial thickness	Yes (*N* = 14)	No (*N* = 88)	2.4 (1.01–4.5)
<7 mm (*N* = 60)	12 (20%)	48 (80%)	
>7 mm (*N* = 40)	2 (5%)	38 (95%)	0.034
	**Thick/irregular endometrium**		
Adenomyosis	Yes (*N* = 10)	No (*N* = 90)	2.5 (1.4–15.9)
Yes (*N* = 11)	6 (54.6%)	5 (45.4%)	
No (*N* = 89)	4 (4.5%)	85 (95.5%)	0.001

*Fisher’s exact test. *P*-value < 0.05*.

## 4 Discussion

In this study, 100 infertile women who met our inclusion criteria were examined to determine the comparison and relationship of hysteroscopy findings and pathology with TVS color Doppler findings of the uterus in the follicular phase. Abnormal hysteroscopy findings were observed in 69% of patients, and this rate reached 73% in pathological examinations. Endometrial polyps (31%) and intrauterine adhesions (10%) were the most common disorders of hysteroscopy findings, while disordered proliferative endometrium (29%) and anovulatory endometrium (17%) were the most common pathological disorders. Also, a TVS color Doppler of the uterus was performed on all patients. Abnormal findings were observed in 66% of cases, and endometrial polyp (25%) was among the most common. The important findings of the present study were the relation between hysteroscopy findings and vaginal color Doppler ultrasound findings.

Hysteroscopy, transvaginal ultrasonography scan, and color Doppler can all be used as screening tests to evaluate the uterine cavity before IVF, especially after unsuccessful IVF. When an abnormality in the uterine cavity is suspected, hysteroscopy is the preferred diagnostic and treatment method, especially when a biopsy is taken from the target tissue and sent to the laboratory for final diagnosis [15,16,17]. This device has very high diagnostic accuracy and is also known for the practical and safe treatment of patients and for restoring fertility [18,19]. Since the strength of its therapeutic effect is related to the type of intrauterine pathology, recent studies show that hysteroscopy is the best minimally invasive method for treating isthmocele. So, the symptoms of all patients have been resolved, and the fertility rate in these patients has been reported as 56.3% [20]. In another study, its very high therapeutic effect on fertility results in uterine arteriovenous malformation patients has been mentioned. And yet, hysteroscopic treatment is only recommended for patients who are hemodynamically stable and who are not bleeding profusely [18].

Hysteroscopy endometrial scratching is another method used for implantation in infertile women. The new trend, the hysteroscopic endometrial fundal incision (EFI), has become one of the most controversial and interesting procedures in the field of reproduction. In this method, damage caused to the endometrium fundal during hysteroscopy could enhance reproductive results and live birth rates in oocyte recipients after implantation failure by regenerating new tissue [21,22]. In general, the mechanisms of the positive effect of endometrial scratching may include: (i) Increasing the endometrial inflammatory response; (ii) Encouraging decidualization; (iii) Increasing the synthesis of growth factors, cytokines, interleukin-11, macrophages, and dendritic cells; and (iv) Increasing the expression of specific endometrial genes [23,24]. In comparison, the importance of EFI for implantation can be justified by three possible reasons. First, unlike the pipelle, where the blind catheter scratches the anterior or posterior uterine wall and never the fundus itself, in this technique, the damage is directed to the fundus, and the depth of the damage is controllable by the surgeon. Second, in cases with an arcuate uterus (type U2a), scraping is simultaneously therapeutic because it restores this congenital variation of the uterine fundus, which was previously considered physiological, without affecting implantation. Third, this method can even detect a percentage of minor abnormalities that would have remained undetected in the group without hysteroscopy [25].

Many studies have been reported to investigate intrauterine abnormalities by hysteroscopy in RIF patients. According to previous studies, intrauterine pathologies in women with RIF diagnosed by hysteroscopy have been reported in 44/9, 52, 37.13, 51.2, and 39.1%, respectively, and the endometrial polyp was usually the most common hysteroscopy abnormality identified in these patients [16,26,27,28,29]. In this study, the rate of abnormal uterine findings with hysteroscopy was somewhat higher than in previous studies. The study content, the average age, and the duration of infertility in these patients may be the main reasons for this study. Additionally, the hysteroscopic findings from a prospective cohort interventional study by Makled et al., which assessed cases of unexplained infertility using diagnostic hysteroscopy and transvaginal sonography, showed that 31% were diagnosed with endometrial polyps, 15% with endometrial hyperplasia, 14% with endometritis, 7% with intrauterine synechiae, 6% with submucous myomas, 6% with cervical stenosis, 7% with congenital uterine anomalies, and 14% of patients had no uterine abnormalities [30]. The current study observed abnormal findings with hysteroscopy in 69% of patients. These findings included endometrial polyp (31%), intrauterine adhesions (10%), uterine septum (8%), submucosal myoma (6%), arcuate uterus (6%), and thick/irregular endometrium (10%). Our study’s prevalence of hysteroscopic findings is somewhat consistent with the above findings.

However, with hysteroscopy, physicians use other tools, such as ultrasonography, for comparison and further evaluation to help diagnose intrauterine pathology [31]. Transvaginal sonography (TVS) is the first-line imaging technique that allows for accurate assessment of the uterus and ovaries, the presence or absence of endometrioma or tubal pathology, the vagina, particularly the areas of the posterior and lateral vaginal fornices, and the rectovaginal septum. Intrauterine parameters, including adenomyosis, can be observed with two-dimensional (2D) TVS showing the typical myometrial features. Although the specificity and sensitivity of TVS in predicting adenomyosis and deeply infiltrating endometriosis (DIE) is high, their evaluation by TVS requires a high level of experience [32,33].

Three-dimensional transvaginal sonography (3D TVS) has also been suggested for assessing posterior sites of DIE without bowel involvement, which improves the diagnostic accuracy of 2D ultrasound [34]. Reports indicate that TVS has poor accuracy in diagnosing vaginal endometriosis [35]. One research team reported that 3D intrauterine ultrasound is an effective method for diagnosing and identifying endometriosis in rectovaginal septum (RVS). However, only a few studies show that 3D ultrasound is better than 2D ultrasound in diagnosing DIE [36,37,38].

Transvaginal four-dimensional hysterosalpingo-contrast sonography (TVS 4D-HyCoSy) is a diagnostic tool for infertility. This device provides the contrast material inside the pelvic area, uterine cavity, and fallopian tubes. It provides the possibility of a 3D representation of the morphology and the path of the fallopian tube opening [39]. Transvaginal two-dimensional hysterosalpingo-contrast sonography (TVS 2D-HyCoSy) and transvaginal three-dimensional hysterosalpingo-contrast sonography (TVS 3D-HyCoSy) are widely considered to be noninvasive, accurate, safe, and reproducible [40]. However, it is not possible to use TVS 2D-HyCoSy to visualize the entire fallopian tube in one plane, and the effectiveness of this method is highly related to sufficient skill [41]. Although 3D-HyCoSy can access the entire fallopian tube, it provides limited information [41]. In contrast, the 4D-HyCoSy can prevent the abovementioned shortcomings in examining infertile women [42,43]. 2D and 3D scanning can also be performed immediately after 4D-HyCoSy to obtain additional diagnostic information.

In general, based on previous studies, RIF patients usually have an endometrial thickness of <7 mm, non-three-layer endometrial pattern, heterogeneous myometrium, uterine artery PI higher than 3, elimination of end-diastolic flow, and endometrial vascularity are reduced compared to healthy people, that the results of examining the findings of our study in RIF patients are also consistent with the above findings [44]. In one study, 789 women with recurrent pregnancy loss (RPL) or RIF were examined to determine the pathological findings of transvaginal ultrasonography in the follicular phase. Most of the patients (69%) did not have obvious abnormal findings in transvaginal ultrasound, but one-third of them (31%) showed uterine pathological findings. These findings in patients include endometrial polyps (10.6%), asherman’s syndrome (1.3%), endometrial hyperplasia (1.1%), arcuate uterus (11%), septate uterus (4.9%) and submucosal myoma (1.6%) [45,46]. While the overall rate of intrauterine pathologies detected by 3D TVS in infertile women was reported to be 15.58% in a recent study [31], our study found abnormal findings in 66% of cases, which included endometrial polyps (25%), intrauterine adhesions (0%), uterine septum (5%), submucosal myoma (6%), arcuate uterus (4%), thick or irregular endometrium (8%), and adenomyosis (11%). In this study, the rate of abnormal findings was higher than in the above study. This increase may be partly due to the differences in the content of these two studies; in our study, only RIF patients were examined, and it is considered one of the strengths of the study. While in the studies mentioned above, both intrauterine insemination and RPL were included or generally examined in infertile women alongside RIF. Also, the abnormal findings of ultrasonography and hysteroscopy were compared in the present study. These two methods usually overlap in the detection of abnormal findings, and this is consistent with previous studies [31,46].

Recently, the effects of adenomyosis on fertility have received much attention. Some of the studies suggest that the presence of adenomyosis does not significantly affect implantation, clinical pregnancy, or live birth rates. However, the RPL is significantly increased in women with adenomyosis. Among the adenomyosis types, diffuse adenomyosis in the junctional zone (JZ) and severe adenomyosis were associated with a significantly increased risk of miscarriage. In contrast, features of adenomyosis exclusively detected in the outer myometrium were associated with persistent pregnancy rates [33]. In support of the effect of adenomyosis on the miscarriage rate, other studies show that interrupted or irregular JZ is a promising predictor of pregnancy loss associated with adenomyosis [47]. These studies demonstrate the importance of examining ultrasound and accurate adenomyosis classification in evaluating and managing patients with RPL. In addition to that, recent studies on the biological basis of the adverse effect of adenomyosis on fertility state the change in endometrial acceptance and disruption in the regulation of hormonal metabolism, which leads to a hyperestrogenic environment [48].

Studies have shown that endometrial receptivity can be predicted and evaluated through the thickness and shape of the endometrium using vaginal color Doppler ultrasonography [49]. Some studies believe that endometrial blood flow is nearly correlated with endometrial receptivity and is helpful for embryo implantation and development [50]. Altogether, the findings show that endometrial patterns can predict the pregnancy results of RIF women and can be used as an essential indicator of endometrial reception in RIF patients [13]. Also, recent studies proved that the vascularity of the endometrium in the repeated implantation failure group is significantly lower than in the pregnancy group [51]. In this study, to improve the prediction and diagnostic power of pathological findings in RIF patients, the relationship between hysteroscopy findings and vaginal color Doppler ultrasonography findings was examined. The important findings of the present study were the significant relation between hysteroscopy findings (submucosal myoma and endometrial polyps) and vaginal color Doppler ultrasound findings (myometrial echogenicity). Also, intrauterine adhesion, endometrial atrophy, and thick/irregular endometrium in hysteroscopy findings were significantly related to uterine thickness, uterine artery parameters, and adenomyosis in vaginal color Doppler ultrasound findings, respectively.

While in other uterine parameters, there was no relation between hysteroscopy findings and vaginal color Doppler ultrasonography findings. This study shows that all subjects with submucosal myoma and endometrial polyps had heterogeneous myometrium. Also, all subjects with intrauterine adhesions had uterine artery PI higher than 3, and most subjects with atrophic endometrium and thick/irregular endometrium were associated with endometrial thickness of less than 7 mm and adenomyosis, respectively. Therefore, by examining the relationship between abnormal uterine findings using these two diagnostic tools, even with the availability of only one of the above tools, it may be possible to increase the estimation, prediction, and etiology of some intrauterine abnormalities in RIF patients. As a result, these findings can be considered strengths of our study in estimating and predicting abnormal uterine findings.

## 5 Conclusion

Considering that this study was conducted only on RIF patients, and they had a high average age with a long history of infertility, therefore we had a limited number of eligible patients, and more abnormal findings were observed in these patients. The findings of vaginal color Doppler ultrasound and hysteroscopy showed an acceptable overlap, and comparing the association between these abnormal findings may be useful in improving the diagnostic power and timely treatment, as well as in revealing possible causes and predicting uterine abnormalities in these patients. Given that these patients require additional examinations, vaginal color Doppler ultrasonography is also recommended alongside hysteroscopy evaluation to enhance diagnostic and therapeutic measures as effectively as possible.

## References

[1] Ma J, Gao W, Li D. Recurrent implantation failure: A comprehensive summary from etiology to treatment. Front Endocrinol. 2023;13:1061766. 10.3389/fendo.2022.1061766 PMC9849692 36686483

[2] Simionescu G, Doroftei B, Maftei R, Obreja B-E, Anton E, Grab D, et al. The complex relationship between infertility and psychological distress. Exp Ther Med. 2021;21:1. 10.3892/etm.2021.9737 PMC7885086 33717249

[3] Borumandnia N, Majd HA, Khadembashi N, Alaii H. Worldwide trend analysis of primary and secondary infertility rates over past decades: A cross-sectional study. Int J Reprod Biomed. 2022;20:37. 10.18502/ijrm.v20i1.10407 PMC8902794 35308328

[4] Abangah G, Rashidian T, Nasirkandy MP, Azami M. A meta-analysis of the prevalence and etiology of infertility in Iran. Int J Fertil Steril. 2023;17:160. 10.22074/IJFS.2023.541991.1215 PMC10189156 37183842

[5] Shingshetty L, Cameron NJ, Mclernon DJ, Bhattacharya S. Predictors of success after in vitro fertilization. Fertil Steril. 2024;121(5):742–51. 10.1016/j.fertnstert.2024.03.003 38492930

[6] Hou J-H, Lu B-J, Huang Y-L, Chen C-H, Chen C-H. Outpatient hysteroscopy impact on subsequent assisted reproductive technology: A systematic review and meta-analysis in patients with normal transvaginal sonography or hysterosalpingography images. Reprod Biol Endocrinol. 2024;22:18. 10.1186/s12958-024-01191-0 PMC10832084 38302947

[7] Lin F, Chen C, Li M, Shi H, Xu X, Jiang X, et al. Complex uterine cavity abnormalities increase the risk of miscarriage in in vitro fertilization/intracytoplasmic sperm injection in fresh cycle-assisted pregnancies. J Minim Invasive Gynecol. 2022;29:891–904. 10.1016/j.jmig.2022.04.010 35490938

[8] Tiwari A, Singh BK, Mishra A. A comparative study to evaluate diagnostic accuracy and correlation between saline infusion sonography, hysterosalpingography and diagnostic hysterolaparoscopy in infertility. Int J Reprod Contracept Obstet Gynecol. 2020;9:670.

[9] Liu Z, Geng Y, Huang Y, Hu R, Li F, Song Y, et al. Letrozole compared with clomiphene citrate for polycystic ovarian syndrome: A systematic review and meta-analysis. Obstet Gynecol. 2023;141:523–34. 10.1097/AOG.0000000000005070 36735392

[10] Wang W-Q, Chu G-H, Hou X-X. A comparison of Doppler measures of ovarian blood flow between women with and without ovarian dysfunction and correlations of Doppler indices with ovarian dysfunction markers: A meta-analysis. Ann Transl Med. 2023;11. 10.21037/atm-22-5813 PMC9929747 36819581

[11] Wu J, Zhu X, He J, Ye C, Pang B, Zhao T, et al. Clinical value of three-dimensional transvaginal ultrasound in diagnosis of endometrial receptivity and ovarian function in patients with infertility. Comput Math Methods Med. 2022;11;2022:8438131. 10.1155/2022/8438131. PMC9117042 35602346

[12] Zhang C-H, Chen C, Wang J-R, Wang Y, Qian W-P. An endometrial receptivity scoring system basing on the endometrial thickness, volume, echo, peristalsis, and blood flow evaluated by ultrasonography. Front Endocrinol. 2022;13:907874. 10.3389/fendo.2022.907874 PMC9395662 36017318

[13] Tong R, Zhou Y, He Q, Zhuang Y, Zhou W, Xia F. Analysis of the guidance value of 3D ultrasound in evaluating endometrial receptivity for frozen-thawed embryo transfer in patients with repeated implantation failure. Ann Transl Med. 2020;8(15):944. 10.21037/atm-20-5463. PMC7475420 32953744

[14] Aguinaldo LD, Sullivant S, Lanzillo EC, Ross A, He J-P, Bradley-Ewing A, et al. Validation of the ask suicide-screening questions (ASQ) with youth in outpatient specialty and primary care clinics. Gen Hosp Psychiatry. 2021;68:52–8. 10.1016/j.genhosppsych.2020.11.006 PMC7855604 33310014

[15] Carson A, Webster F, Polzer J, Bamford S. The power of potential: Assisted reproduction and the counterstories of women who discontinue fertility treatment. Soc Sci Med. 2021;282:114153. 10.1016/j.socscimed.2021.114153 34171700

[16] Acet F, Sahin G, Goker ENT, Tavmergen E. The effect of hysteroscopy and conventional curretage versus no hysteroscopy on live birth rates in recurrent in vitro fertilisation failure: A retrospective cohort study from a single referral centre experience. J Obstet Gynaecol. 2022;42:2134–8. 10.1080/01443615.2022.2033963 35170394

[17] Vitale SG, Angioni S, Parry JP, Di Spiezio Sardo A, Haimovich S, Carugno J, et al. Efficacy of hysteroscopy in improving fertility outcomes in women undergoing assisted reproductive technique: A systematic review and meta-analysis of randomized controlled trials. Gynecol Obstet Invest. 2023;88:336–48. 10.1159/000534794 PMC10794974 37899034

[18] Calzolari S, Cozzolino M, Castellacci E, Dubini V, Farruggia A, Sisti G. Hysteroscopic management of uterine arteriovenous malformation. J Soc Laparoendosc Surg. 2017;21:e2016.00109. 10.4293/JSLS.2016.00109 PMC5385144 28439193

[19] Calzolari S, Cozzolino M, Castellacci E. Uterine arteriovenous malformation: Hysteroscopic identification is possible. J Minim Invasive Gynecol. 2016;23:293–4. 10.1016/j.jmig.2015.10.007 26498539

[20] Calzolari S, Sisti G, Pavone D, Ciocia E, Bianchini N, Cozzolino M. Prevalence of infertility among patients with isthmocele and fertility outcome after isthmocele surgical treatment: a retrospective study. Ochsner J. 2019;19:204–9. 10.31486/toj.18.0048 PMC6735591 31528130

[21] Peitsidis N, Tsakiridis I, Najdecki R, Michos G, Kalogiannidis I, Athanasiadis A, et al. Hysteroscopic endometrial fundal incision versus hysteroscopy only in oocyte recipients: a randomized controlled trial assessing the reproductive outcomes. Int J Fertil Steril. 2024;18:3. 10.22074/IJFS.2024.2009369.1523 PMC11263851 39033364

[22] Peitsidis N, Tsakiridis I, Najdecki R, Michos G, Chouliara F, Timotheou E, et al. Diagnostic hysteroscopy with endometrial fundal incision may improve reproductive outcomes in oocyte recipients after implantation failure. JBRA Assist Reprod. 2023;27:689. 10.5935/1518-0557.20230037 PMC10718553 37962971

[23] Gnainsky Y, Granot I, Aldo PB, Barash A, Or Y, Schechtman E, et al. Local injury of the endometrium induces an inflammatory response that promotes successful implantation. Fertil Steril. 2010;94:2030–6. 10.1016/j.fertnstert.2010.02.022 PMC3025806 20338560

[24] Chen T, Shi H, Fang LL, Su YC. The effect of endometrial injury on reproductive outcomes of frozen–thawed embryo transfer cycles in women with one implantation failure. J Int Med Res. 2020;48:0300060520913130. 10.1177/0300060520913130 PMC7133396 32216503

[25] Najdecki R, Peitsidis N, Tsakiridis I, Michos G, Timotheou E, Chartomatsidou T, et al. Hysteroscopic endometrial fundal incision in oocyte recipients before embryo transfer may improve reproductive outcomes: A prospective study. Int J Fertil Steril. 2023;18:40. 10.22074/IJFS.2023.560746.1354 PMC10692741 38041458

[26] El-Sheikh A, Abou Senna H, Hussein M. Role of hysteroscopic guided endometrial biopsy in diagnosis of endometrial pathology in patients with unexplained recurrent implantation failure. Al-Azhar Med J. 2020;49:775–84.

[27] Gao M, Sun Y, Xie H, Fang S, Zhao X. Hysteroscopy prior to repeat embryo transfer may improve pregnancy outcomes for asymptomatic women with repeated implantation failure. J Obstet Gynaecol Res. 2015;41:1569–76. 10.1111/jog.12773 26223364

[28] Pabuçcu EG, Yalçın İ, Bodur T, Çağlar GS, Pabuçcu R. Impact of office hysteroscopy in repeated implantation failure: Experience of a single center. J Turkish Ger Gynecol Assoc. 2016;17:197. 10.5152/jtgga.2016.16166 PMC5147758 27990088

[29] Al-Turki HA. Hysteroscopy as an investigation tool in recurrent implantation failure in vitro fertilization. Saudi Med J. 2018;39:243. 10.15537/smj.2018.3.21379 PMC5893912 29543301

[30] Abdellah AH, Ali AE-NAE-G, Fadel SA, Hashem H, Taha ASAM. Hysteroscopic endometrial biopsy after intracytoplasmic sperm injection failure for women with unexplained infertility. SVU-Int J Med Sci. 2024;7:592–9.

[31] Naredi N, Sharma R, Gurmeet P. Can three-dimensional transvaginal sonography replace office hysteroscopy in detecting uterine abnormalities in infertility patients? J Hum Reprod Sci. 2021;14:392. 10.4103/jhrs.jhrs_97_21 PMC8812396 35197685

[32] Exacoustos C, Zupi E, Piccione E. Ultrasound imaging for ovarian and deep infiltrating endometriosis. Semin Reprod Med. 2017;35(1):5–24. 10.1055/s-0036-1597127 28076877

[33] Cozzolino M, Cosentino M, Loiudice L, Martire FG, Galliano D, Pellicer A, et al. Impact of adenomyosis on in vitro fertilization outcomes in women undergoing donor oocyte transfers: A prospective observational study. Fertil Steril. 2024;121:480–8. 10.1016/j.fertnstert.2023.11.034 38043844

[34] Guerriero S, Alcázar JL, Ajossa S, Pilloni M, Melis GB. Three‐dimensional sonographic characteristics of deep endometriosis. J Ultrasound Med. 2009;28:1061–6. 10.7863/jum.2009.28.8.1061 19643789

[35] Hudelist G, Oberwinkler K, Singer C, Tuttlies F, Rauter G, Ritter O, et al. Combination of transvaginal sonography and clinical examination for preoperative diagnosis of pelvic endometriosis. Hum Reprod. 2009;24:1018–24. 10.1093/humrep/dep013 19202143

[36] Guerriero S, Saba L, Ajossa S, Peddes C, Angiolucci M, Perniciano M, et al. Three-dimensional ultrasonography in the diagnosis of deep endometriosis. Hum Reprod. 2014;29:1189–98. 10.1093/humrep/deu054 24664128

[37] Pascual MA, Guerriero S, Hereter L, Barri-Soldevila P, Ajossa S, Graupera B, et al. Diagnosis of endometriosis of the rectovaginal septum using introital three-dimensional ultrasonography. Fertil Steril. 2010;94:2761–5. 10.1016/j.fertnstert.2010.02.050 20356583

[38] Pascual MA, Guerriero S, Hereter L, Barri-Soldevila P, Ajossa S, Graupera B, et al. Three‐dimensional sonography for diagnosis of rectovaginal septum endometriosis: interobserver agreement. J Ultrasound Med. 2013;32:931–5. 10.7863/ultra.32.6.931 23716513

[39] Briceag I, Costache A, Purcarea V, Cergan R, Dumitru M, Sajin M, et al. Fallopian tubes–literature review of anatomy and etiology in female infertility. J Med Life. 2015;8:129. PMC4392087 25866566

[40] Kupesic S, Plavsic BM. 2D and 3D hysterosalpingo-contrast-sonography in the assessment of uterine cavity and tubal patency. Eur J Obstet Gynecol Reprod Biol. 2007;133:64–9. 10.1016/j.ejogrb.2006.10.010 17329010

[41] Exacoustos C, Di Giovanni A, Szabolcs B, Binder-Reisinger H, Gabardi C, Arduini D. Automated sonographic tubal patency evaluation with three-dimensional coded contrast imaging (CCI) during hysterosalpingo-contrast sonography (HyCoSy). Ultrasound Obstet Gynecol. 2009;34(5):609–12. 10.1002/uog.7442 19852043

[42] Wang Y, Qian L. Three-or four-dimensional hysterosalpingo contrast sonography for diagnosing tubal patency in infertile females: A systematic review with meta-analysis. Br J Radiol. 2016;89:20151013. 10.1259/bjr.20151013 PMC5257311 27109737

[43] Wang W, Zhou Q, Gong Y, Li Y, Huang Y, Chen Z. Assessment of fallopian tube fimbria patency with 4‐dimensional hysterosalpingo‐contrast sonography in infertile women. J Ultrasound Med. 2017;36:2061–9. 10.1002/jum.14244 28543598

[44] Nesbit CB, Blanchette-Porter M, Esfandiari N. Ovulation induction and intrauterine insemination in women of advanced reproductive age: A systematic review of the literature. J Assist Reprod Genet. 2022;39:1445–91. 10.1007/s10815-022-02551-8 PMC9365895 35731321

[45] Alcazar JL, Carriles I, Cajas MB, Costa S, Fabra S, Cabrero M, et al. Diagnostic performance of two-dimensional ultrasound, two-dimensional sonohysterography and three-dimensional ultrasound in the diagnosis of septate uterus—A systematic review and meta-analysis. Diagnostics. 2023;13:807. 10.3390/diagnostics13040807 PMC9955687 36832295

[46] Shiva M, Ahmadi F, Arabipoor A, Oromiehchi M, Chehrazi M. Accuracy of two-dimensional transvaginal sonography and office hysteroscopy for detection of uterine abnormalities in patients with repeated implantation failures or recurrent pregnancy loss. Int J Fertil Steril. 2018;11:287. 10.22074/ijfs.2018.5034 PMC5641460 29043704

[47] Barbanti C, Centini G, Lazzeri L, Habib N, Labanca L, Zupi E, et al. Adenomyosis and infertility: The role of the junctional zone. Gynecol Endocrinol. 2021;37:577–83. 10.1080/09513590.2021.1878131 33587014

[48] Selntigia A, Molinaro P, Tartaglia S, Pellicer A, Galliano D, Cozzolino M. Adenomyosis: An update concerning diagnosis, treatment, and fertility. J Clin Med. 2024;13:5224. 10.3390/jcm13175224 PMC11396652 39274438

[49] Asyura MMAZ, Komariah M, Amirah S, Faisal EG, Maulana S, Platini H, et al. Analysis of varying microRNAs as a novel biomarker for early diagnosis of preeclampsia: A scoping systematic review of the observational study. Int J Prev Med. 2023;14:36. 10.4103/ijpvm.ijpvm_156_22 PMC10284242 37351051

[50] Shaulov T, Sierra S, Sylvestre C. Recurrent implantation failure in IVF: A Canadian fertility and andrology society clinical practice guideline. Reprod Biomed Online. 2020;41:819–33. 10.1016/j.rbmo.2020.08.007 32962928

[51] Bayati F, Eftekhar M, Homayoon N, Fatehi H. Comparison of Doppler ultrasound indices of uterine artery and sub endometrial blood supply in frozen embryo transfer with and without repeated implantation failure: A cross-sectional study. Int J Reprod BioMed. 2023;21:937. 10.18502/ijrm.v21i11.14657 PMC10823118 38292508

